# The Influence of Infection by Different *Leishmania (Viannia) braziliensis* Isolates on the Pathogenesis of Disseminated Leishmaniasis

**DOI:** 10.3389/fcimb.2021.740278

**Published:** 2021-09-10

**Authors:** Walker N. Oliveira, Andreza S. Dórea, Pedro P. Carneiro, Maurício T. Nascimento, Lucas P. Carvalho, Paulo R. L. Machado, Albert Schriefer, Olívia Bacellar, Edgar M. Carvalho

**Affiliations:** ^1^Serviço de Imunologia, Complexo Hospitalar Universitário Prof. Edgard Santos, Universidade Federal da Bahia, Salvador, Brazil; ^2^Instituto Nacional de Ciência e Tecnologia de Doenças Tropicais - INCT-DT (CNPq/MCT), Salvador, Brazil; ^3^Instituto Pesquisa Gonçalo Moniz – Fiocruz-Bahia, Salvador, Brazil; ^4^Departamento de Ciências da Biointeração, Instituto de Ciências da Saúde, Universidade Federal da Bahia, Salvador, Brazil

**Keywords:** cutaneous leishmaniasis, monocytes, inflammatory response, disseminated leishmaniasis, *Leishmania (Viannia) braziliensis*

## Abstract

Disseminated Leishmaniasis (DL) is an emerging and severe form of *Leishmania (Viannia) braziliensis* infection defined by the presence of 10 and up to more than 1,000 skin lesions. The mechanisms underlying parasite dissemination remain unknown. Genotypic differences among species of *L. braziliensis* have been associated with different clinical forms of disease. The present work compared the function of monocytes obtained from patients with cutaneous leishmaniasis (CL) and DL in response to infection with *L. braziliensis* isolates of both these two clinical forms of disease. Mononuclear cells obtained from DL and CL patients were infected with different *L. braziliensis* isolates, and numbers of infected cells, parasite load, respiratory burst, TLR2 and TLR4 expression and cytokine production were evaluated. DL isolates infected more monocytes, induced greater respiratory burst, and more cytokine production compared to isolates from CL patients regardless of the origin of monocytes (DL or CL). However, greater parasite multiplication and higher TLR2 and TLR4 expression were seen in monocytes from DL patients compared to CL following infection with DL isolates. Our results indicate the participation of both parasite genotype and host factors in the pathogenesis of DL.

## Introduction

Disseminated leishmaniasis (DL), a severe form of *Leishmania (Viannia) braziliensis* infection, is defined by the presence of more than 10, and up to 1,000 papular, acneiform and ulcerated lesions across at least two separate parts of the body (Turetz et al., 2002). DL has also been documented in infection by other *Leishmania* species (Calvopina et al., 2005; Alborzi et al., 2008; Rincon et al., 2009; Newlove et al., 2012), and differs from classical cutaneous leishmaniasis (CL), which is characterized by the presence of a single or few well-delimited ulcers with raised borders. Often confused with diffuse CL (DCL), DL is considered clinically, immunologically and histopathologically distinct from DCL (Petersen et al., 1984; Carvalho et al., 1994; Silveira et al., 2004; Christensen et al., 2019). While DCL patients exhibit poor lymphocyte proliferation and absent or low production of IFN-γ upon exposure to soluble leishmania antigen (SLA) (Petersen et al., 1984; Christensen et al., 2019), an impaired Th1 immune response has not been documented in DL (Machado et al., 2011). Cytokine expression at lesion sites is remarkably similar between DL and CL, and no comparative histopathological or immunochemical differences have been evidenced by ulcer tissue analysis (Machado et al., 2011; Dantas et al., 2014). Recently, emphasis has been placed on the role of CD8^+^ T cells in the pathogenesis of *L. braziliensis* infection. CD8^+^ T cells from CL patients were observed to kill leishmania-infected monocytes, yet did not kill parasites (Cardoso et al., 2015). Moreover, transcriptomic analysis of lesions from CL patients revealed the high expression of genes associated with the cytolytic pathway, and CD8 ^(+)^ T cells obtained from lesions exhibited a cytolytic phenotype (Novais et al., 2013). Moreover, disease progression and metastasis in *L. (V.) braziliensis*-infected mice were found to occur independently from parasite burden, instead being directly associated with the presence of CD8 ^(+)^ T cells (Novais et al., 2013).

Macrophages, the main cell type responsible for leishmania killing, also play a role in disease pathology (Scott et al., 1988; Novais et al., 2014). The participation of monocytes is also notable in host inflammatory response against CL, as the enhancement of intermediate monocytes provides an important source of TNF, a cytokine associated with tissue damage in tegumentary leishmaniasis (Passos et al., 2015). The Toll-like receptor (TLR) signaling pathway is a primary defense mechanism against infectious agents (Medzhitov, 2007; Tuon et al., 2008). Monocytes from CL patients express more TLR2, TLR4 and TLR9 *ex vivo* or in soluble leishmania antigen (SLA)-stimulated cultures than cells from healthy subjects (HS) (Vieira et al., 2013; Carneiro et al., 2016). The elevated expression of TLR2 and TLR4 by intermediate monocytes from *L. (V.) braziliensis*-infected CL patients in comparison to HS has been associated with TNF production (Polari et al., 2019). On the other hand, the neutralization of TLR4 was found to decrease TNF production by peripheral blood mononuclear cells (PBMC) infected with *L. (V.) braziliensis* (Galdino et al., 2016). While monocytes from CL patients produce more reactive oxygen species following exposure to *L. (V.) braziliensis* than monocytes from HS (Carneiro et al., 2016), diminished leishmania killing has been observed in CL patients compared to subjects with asymptomatic *L. (V.) braziliensis* infection (Muniz et al., 2016). As monocytes function has not been evaluated in DL, the present study aimed to compare activation and killing capability of DL versus CL monocytes upon exposure to DL and CL isolates. Parasite survival and proliferation in monocytes are elevated by molecules such as superoxide dismutase (SOD) and prostaglandin E2 (PGE2), which are both enhanced in CL and visceral leishmaniasis however, the literature contains no reports on these molecules in DL (Araujo-Santos et al., 2014; Khouri et al., 2014; Saha et al., 2014).

Parasite factors are also known to influence the pathogenesis of leishmaniasis. As *L. (V.) braziliensis* is polymorphic, dissimilarity among strains at locus CHR 28/425451 or CHR 32/1356278 has been associated with different clinical forms of disease, as well as failure to antimonial therapy (Queiroz et al., 2012; Guimaraes et al., 2016; Silva et al., 2018). Supporting the notion that differences among same-species of leishmania may influence immune response, SLA prepared using *L. (V.) braziliensis* isolates from DL patients was found to induce higher TNF production in mononuclear cells from both CL and DL patients than SLA similarly prepared using isolates of *L. (V.) braziliensis* obtained from CL patients (Leopoldo et al., 2006). Moreover, the isolates of *L. (V.) braziliensis* obtained from DL patients were found to be less internalized by neutrophils from HS, and also induced lower oxidative burst as well as reduced expression of neutrophil activation markers (Cardoso et al., 2019). Nonetheless, the literature contains scarce data on monocyte function in DL. The present study endeavored to compare the ability of DL and CL isolates to penetrate and survive in monocytes, as well as to induce molecules related to the pathogenesis of DL.

## Material and Methods

### Patients

A total of 24 patients with DL and 24 with CL were included; all individuals sought medical attention at the Corte de Pedra Health Clinic, located in the municipality of Presidente Tancredo Neves (Bahia-Brazil), between September 2017 and May 2019. For comparison purposes, we included 12 healthy subjects (HS) without exposure to leishmania from a non-endemic area. DL patients presented more than 10 acneiform, popular and ulcerated lesions, along two or more non-contiguous parts of the body (head, trunk, arms or legs). CL patients presented 1-3 ulcers with raised borders. Infection was diagnosed *via* detection of *L. (V.) braziliensis* DNA by PCR in biopsied ulcer tissue samples (Weirather et al., 2011) in conjunction with parasite isolation in culture, or identification by histopathologic analysis. All experiments were performed prior to the administration of therapy.

### Ethics Statement

The present research protocol received approval from the Institutional Review Board of the Federal University of Bahia, and was approved by the Brazilian Commission for Ethics in Research (CONEP) (protocol no.: 2.114.874). All subjects agreed to voluntarily participate and provided a written term of informed consent. All methods were performed in accordance with the guidelines and regulations stipulated by CONEP.

#### Parasites

Species determination was based upon HSP70 PCR-restriction fragment length polymorphism and later confirmed by serial real-time quantitative PCR (Weirather et al., 2011). The genotyping of *L*. (*V*.) *braziliensis* recovered from CL and DL lesions was performed as previously described (Schriefer et al., 2004; Queiroz et al., 2012). CL and DL isolates were genotyped according to haplotypes of polymorphic nucleotides at locus CHR28/455451, which was previously shown to distinguish among *L. (V.) braziliensis* strains (Queiroz et al., 2012). CL and DL *L. (V.) braziliensis* isolates were initially cultured in biophasic Novy-MacNeal-Nicolle medium with liver infusion tryptose at 26°C for 1-2 weeks, and then used to infect monocytes. Cellular suspensions were transferred to Schneider’s medium containing 10% heat-inactivated fetal calf serum and 2 mM l-glutamine, then reincubated at 26°C for up to two weeks. Parasites were frozen in liquid nitrogen without any further subculturing in 10% dimethyl sulfoxide with 90% growth medium, and finally thawed prior to use in experimentation.

### Soluble Leishmania Antigen

Soluble *Leishmania* antigen (SLA) was prepared from *L. (V.) braziliensis* isolated (MHOM/BR/2001) from a CL patient and with a DL isolate, as previously described (Reed et al., 1986). The antigen was tested for endotoxins using the Limulus amebocyte lysate test, and used at a concentration of 5 μg/mL.

### Isolation of Human Peripheral Blood Cells and Infection With *L. (V.) braziliensis* Isolates

Peripheral blood mononuclear cells (PBMC) were isolated from DL and CL patients and HS; PBMCs were used in all experiments. Heparinized venous blood was layered (Bacellar et al., 2002) and resuspended in RPMI 1640 medium supplemented with 5% fetal calf serum and antibiotics (GIBCO BRL, Grand Island, NY USA). PBMCs (1x10^6^ cells/tube) were infected with stationary-phase *L. (V.) braziliensis* isolates at a ratio of 5:1. After 2 hours of infection at 37°C under 5% CO_2_, extracellular parasites were removed following centrifugation. Cells were placed in complete RPMI 1640 medium and incubated for an additional 48 hours. Finally, the numbers of infected cells and intracellular parasites were determined by microscopic evaluation of 100 monocytes following Romanowsky staining of cytocentrifuge preparations.

### Evaluation of Parasite Viability

After 48 hours of infection with *L. (V.) braziliensis* isolates, PBMCs were washed and the medium was replaced with 0.5 ml of Schneider’s medium (Sigma-Aldrich) supplemented with 10% fetal calf serum. Cells were then cultured at 26°C for an additional 48 h. Viable parasites was determined by counting extracellular motile promastigotes using a hemocytometer (Novais et al., 2009).

### Determination of PGE2 and SOD

PGE2 production was evaluated using reagents purchased from R&D Systems (Minneapolis, USA) in the supernatants of cultured PBMCs (3x10^6^ cells/mL) stimulated with SLA (5µg/mL). Results are expressed in pg/mL. SOD serum levels were determined using a Human Cu/Zn Superoxide Dismutase ELISA kit (ABCAM. Cambridge Science Park, UK).

### Evaluation of Oxidative Burst

PBMCs (1x10^6^) were incubated with 10 ng/mL dihydrorhodamine 123 (DHR-FITC) (Cayman Chemical Company) for 10 minutes at 37°C under 5% CO_2_. Cells were then exposed to *L. (V.) braziliensis* (ratio 5:1) for 25 minutes (37°C, 5% CO_2_). Phorbol 12-myristate 13-acetate (PMA-Invivogen) at 1µg/mL was used as positive control. Monocytes were stained for anti-CD14 surface markers (APC clone M5E2, BD Pharmingen) and quantified by nonspecific fluorescence using forward scatter (FSC) and side scatter (SSC) parameters to determine cell size and granularity, respectively. Cells were then gated based on CD14 expression and DHR 123 oxidation (**Figure 3**). A total of 200,000 cells per tube were evaluated on a FACS Canto II flow cytometer (BD); data analysis was performed using FlowJo software (Tree Star Inc).

### Monocyte Expression of TLR2 and TLR4

Monocyte surface expression of TLR2 and TLR4 were analyzed by median of the MFI, both *ex vivo* and *in vitro* after infection with *L. (V.) braziliensis* for 2 hours. The following antibodies were used: APC-conjugated anti-CD14 (APC clone M5E2, BD Pharmingen); PE-conjugated anti-TLR2 (clone TL2.1); PE-conjugated anti-TLR4 (clone HTA125) (eBioscience, San Diego, CA, USA). A total of 200,000 cells per tube were evaluated on a FACS Canto II flow cytometer (BD); data analysis was performed using FlowJo software (Tree Star Inc).

### Evaluation of Cytokine Production

PBMCs were either infected with different isolates (CL or DL) of *L. (V.) braziliensis* or left uninfected. To perform flow cytometry, 1x10^6^ PBMCs were stained with a fluorochrome-conjugated CD14 surface marker antibody (APC clone M5E2, BD Pharmingen) and fixed in 2% formaldehyde. For intracellular staining, fixed cells were permeabilized using a cytofix/cytoperm kit (BD-Bioscience) and stained intracellularly with PE-conjugated anti-TNF (clone Mab11, eBioscience), PE-conjugated anti-CXCL9 (clone B8-11, BD Biosciences) and PE-conjugated anti-CXCL10 (clone J034D6, BioLegend) antibodies. A total of 200,000 cells per tube were evaluated on a FACS Canto II flow cytometer (BD); data analysis was performed using FlowJo software (Tree Star Inc).

### Evaluation of Cytokine Production by ELISA

PBMCs (3x10^6^) were stimulated with 5µg of SLA from CL or DL (37°C, 5% CO_2_) for 72 hours. Supernatants were collected and stored at -70°C until the time of analysis. CXCL10, TNF and CXCL9 production was evaluated by ELISA (R&D Systems), performed according to the manufacturer’s instructions; results are expressed in pg/mL.

### Statistical Analysis

Categorical variables with normal distribution were analyzed using the Student’s T test. Comparisons between different isolates within a single group were performed using Wilcoxon’s signed-rank test, while comparisons between groups were performed using the nonparametric Mann-Whitney U test. Differences among three or more groups were assessed by analysis of variance (Kruskal-Wallis) with Dunn’s post-test. Statistical significance was considered when p <0.05. The graphs were generated by statistical software Prism GraphPad version 8.0.1.244. Results are expressed as median values and interquartile range.

## Results

**Figure 1:**
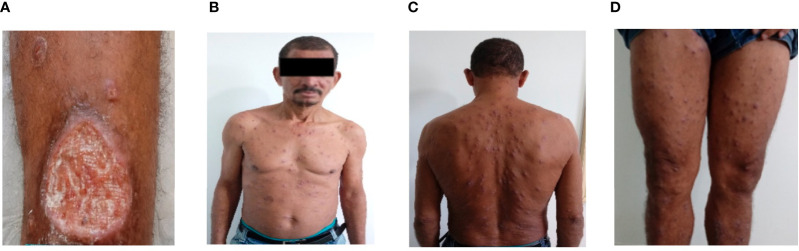
Disseminated Leishmaniasis. Picture of a 55 years old man who initially presented an ulcer (55X45mm) in a left leg **(A)**. After 30 days he suddenly had fever, chills and 485 acneiform and papular lesions were documented in the face, trunk and superior and inferior limbs **(B–D)**.

The demographic and clinical features of the 24 DL and 24 CL patients included in the study are shown in **Table 1**. No differences were detected with regard to age, gender or size of the largest lesion. As expected, higher numbers of lesions were found in DL patients compared to CL, in addition to longer illness duration and a greater occurrence of mucosal disease. The **Figure 1** shows a case of a DL patient who initially had only one ulcer and after 30 days of the disease suddenly appeared hundreds of lesions spread in the body.

**Table 1:** Clinical and epidemiological characteristics of patients with disseminated leishmaniasis (DL) and cutaneous leishmaniasis (CL).

	Disseminated Leishmaniasis (n = 24)	Cutaneous Leishmaniasis (n = 24)	*p value*
Age, media ± standard deviation	36 ± 13	37 ± 14	0.71
Gender, Males (%)	21 (87.5%)	17 (70.8%)	0.28
Number of lesions, Median (IQ)	30.5 (13-800)	2 (1-7)	<0.0001
Duration of lesion, Median (IQ)	60 (26-120)	32 (15-90)	<0.001
Size of largest lesion mm2, Median (IQ)	315 (25-2750)	225 (25-1600)	0.74
Presence of mucosal lesion	7/24 (29%)	0	0.009

### Ability of DL and CL *L. (V.) braziliensis* Isolates to Infect Monocytes from DL and CL Patients

**Figure 2:**
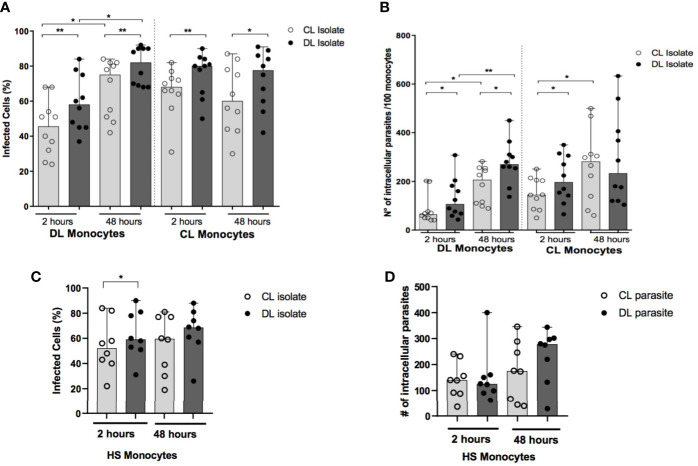
Frequency of infected monocytes and parasite load after infection with different isolates of *L. braziliensis*: PBMC from patients with DL (n=12),CL (n=12) and HS (n=8) were infected with different isolates of *L. braziliensis* at a ratio of 5:1 for 2 h or 48 h. The number of infected cells **(A, C)** and the number of intracellular parasites **(B, D)** were evaluated by optical microscopy in 100 monocytes following Romanowsky staining. P values were calculated using Wilcoxon’s signed-rank for analyses within the same clinical form, while comparisons between different clinical forms were assessed using the Mann-Whitney test (*p < 0.05) (**p < 0.01).

Similar numbers of amastigotes are seen in biopsied ulcer samples from DL and CL patients (Dantas et al., 2014). However, multiple lesions in DL patients imply greater total numbers of parasites compared to CL. Here we infected monocytes obtained from DL and CL patients with an isolate of DL or CL (**Figure 1**). Regardless of donor cell origin, we identified higher frequencies of infected cells and greater numbers of amastigotes per 100 cells in monocytes infected with an isolate from DL compared to CL at both 2 and 48 hours after infection (**Figures 2A, B**). The median frequency of DL monocytes infected with a CL isolate was 46% (30-58 cells) at 2 hours *versus* 58% (45-76 cells) for a DL isolate (p<0.01), compared to monocytes from CL patients: 45% (24-70 cells) for the CL isolate compared to 54% (37-84 cells) for the DL isolate (p<0.001). Similar patterns were observed at 48 hours, yet a higher frequency of DL monocytes infected with the DL isolate was observed after 48 hours of infection in comparison to the earlier timepoint. With respect to HS cells, the frequency of monocytes infected with the DL isolate was higher at 2 hours (p<0.05), but not at 48 hours, compared to the CL isolate (**Figures 2C, D**).

Regarding parasite load after 2 hours of infection, the number of parasites in DL monocytes infected with the CL isolate was 64 (range: 48-101) amastigotes/100 cells *versus* the DL isolate: 107 (range: 66-188) amastigotes/100 cells (p<0.05). The number of amastigotes/100 cells was also higher in CL monocytes infected with the DL isolate compared to CL (P<0.05) at this same timepoint. Again, higher numbers of intracellular parasites were seen in DL monocytes after 48 hours of infection compared to the earlier timepoint. Difference was only noted in HS monocytes at 48 hours of infection, when higher number of amastigotes in cells infected with DL was found in comparison to the CL isolate.

### Viability of Extracellular Promastigotes

**Figure 3:**
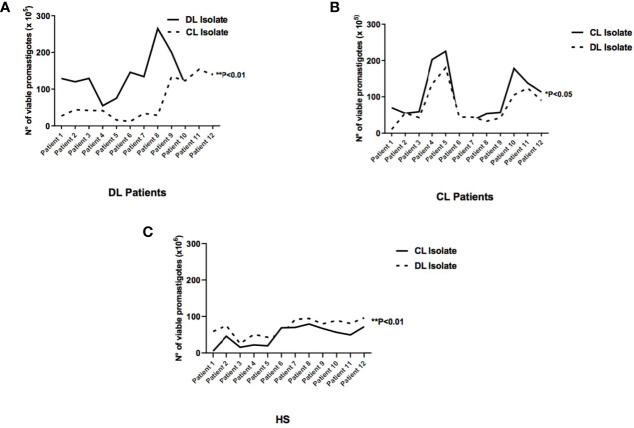
Viability of extracellular promastigotes in the supernatants of cultured monocytes from patients with DL, CL or healthy subjects following infection with different *L. braziliensis* isolates: PBMC from patients with DL (n=12), CL (n=12) or HS (n=12) were infected with different isolates of *L. braziliensis* (DL or CL) for 48 hours. After this period RPMI medium was replaced by Schneider culture medium for another 48 hours. The number of viable promastigotes was evaluated by optical microscopy. **(A)** Monocytes from patients with DL; **(B)** Monocytes from patients with CL; **(C)** Monocytes from HS. Results are expressed as median values and statistical analysis was performed using the Wilcoxon’s signed-rank test. Comparisons between groups were performed using the Mann-Whitney statistical test (*p < 0.05) (**p < 0.01).

Parasite viability was evaluated in the supernatants of cell cultures 48 hours after infection to determine whether the higher frequency of infected cells and elevated number of parasites detected in monocytes infected with DL isolates could lead to cell lysis and, consequently, the release of promastigotes in the supernatant of monocyte cultures (**Figure 3**). Higher numbers of parasites were quantified in the supernatants of DL monocytes when isolates obtained of DL patients were used 129 (55-265) compared with CL isolates 64 (22-205), (p<0.05). There were no differences in the number of viable promastigotes in supernatants of CL or HS monocytes infected with *L. (V.) braziliensis* from DL versus CL (**Figures 3A–C**). These results indicate that in response to *L. (V.) braziliensis* infection, DL monocytes presenter a decreased capacity to kill *Leishmania* compared to CL monocytes.

### Serum SOD Levels and PGE2 Production by DL Monocytes

Increased SOD and PGE2 production in patients with CL has been associated with higher parasite burden (Schriefer et al., 2004; Khouri et al., 2009). While no differences were seen in systemic SOD production between DL (184 pg/mL; range: 130-263 pg/mL) and CL patients (256 pg/mL; range: 139-411 pg/mL), SOD levels were higher (P<0.001) than those found in HS (26 pg/mL; range: 7-77pg/mL). DL monocytes were also observed to produce higher levels of PGE2 (461 pg/mL; range: 0-1443) in the absence of SLA stimulation compared to CL monocytes 0 pg/mL; 0-620pg/mL). Furthermore, under SLA stimulation DL monocytes produced higher levels of PGE2 (747 pg/mL; range: 0-2186 pg/mL) than monocytes from CL patients (0 pg/mL; range: 0-5313 pg/mL).

### Oxidative Burst

**Figure 4:**
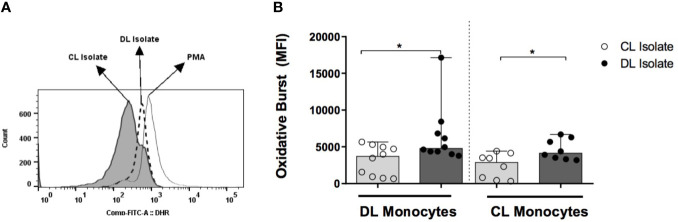
Induction of oxidative burst by monocytes from patients with DL, CL or healthy subjects following infection with different *L. braziliensis* isolates: PBMC from patients with DL (n=12), CL (n=12) or HS (n=8) were treated with DHR (10ng/mL; 10 min) and infected with *L. braziliensis* isolates from DL or CL patients for 25 minutes at a ratio of 5:1 cells. PMA (1ug/ml) was used as positive control. Cells were stained with anti-CD14 for flow cytometric evaluation. **(A)** Representative gating strategy detailing CD14+ and DHR expression in monocytes from a CL patient. **(B)** Data representative as median of the mean fluorescence intensity (MFI) of oxidative burst induction in DL and CL monocytes infected with different isolates. All p values were obtained using Wilcoxon’s signed-rank testing. Comparisons made between groups using Mann- Whitney statistical testing. (*p < 0.05).

To determine whether the induction of respiratory burst differed in parasites obtained from DL and CL patients, and to determine the impairment of this capability in DL monocytes, DL, CL and HS monocytes were infected with isolates from DL and CL patients. In monocytes from DL patients infected with a CL isolate, median levels of dihydrorhodamine (DHR) expression, represented by mean fluorescence intensity (MFI), were 3715 (730-5310) *versus* 4790 (4000-8430) when infected with a DL isolate (P<0.05). DHR expression in CL monocytes infected with a CL isolate was 2870 (330-4400) *versus* 4140 (3180-6700) with DL P<0.05 (**Figure 4**). No differences in DHR expression were seen between DL and CL monocytes infected with either type of isolate. In HS cells, infection with the DL isolate also induced higher oxidative burst compared to CL.

### Expression of TLR2 and TLR4 in Monocytes Infected With DL and CL Isolates

**Figure 5:**
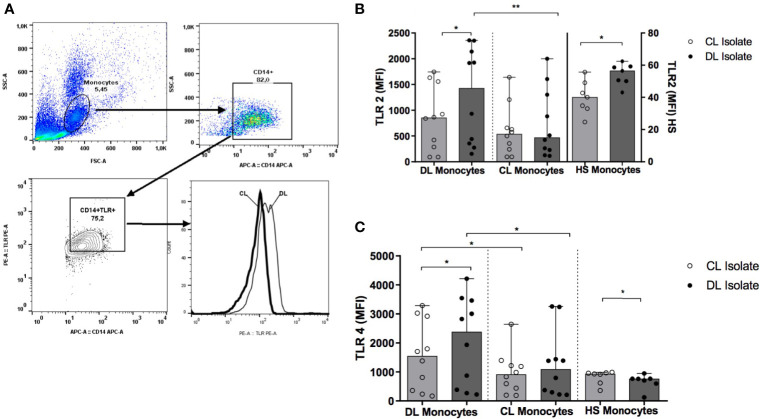
TLR2 and TLR4 expression in monocytes from DL and CL patients. PBMC from DL (n=12), CL (n=12) and HS (n= 7) patients were infected with different isolates of *L. braziliensis* at a ratio of 5:1 for 2 h Following stimulation, monocytes were marked with anti-CD14 antibodies, and with anti-TLR2 or anti-TLR4 for flow cytometry analysis. **(A)** Figure representative of flow cytometry gating strategy; **(B)** Expression of TLR2 **(C)** Expression of TLR4. Data representative with median of mean fluorescence intensity (MFI) values. All p values were obtained using Wilcoxon’s signed-rank testing; comparisons between groups made using the Mann- Whitney statistical test. (*p < 0.05) (**p < 0.01).

We found no differences in the *ex vivo* expression of TLR2 and TLR4 when comparing monocytes from DL and CL patients (data not shown). **Figure 5** illustrates TLR2 and TLR4 expression after infection with each *L. (V.) braziliensis* isolate. Median TLR2 and TLR4 expression levels are represented as MFI. While monocytes from DL patients expressed elevated levels of TLR2 and TLR4 following infection with a DL isolate compared to CL: 1425 (274-2350) and 2384 (270-3542) *versus* 853 (91-1633) and 1544 (235-3028), respectively (P<0.05), similar expression was observed for both receptors when DL or CL isolates were used to infect CL monocytes (P>0.05). Additionally, TLR2 and TLR4 expression was higher in DL monocytes compared to CL monocytes after infection with a DL isolate (p<0.01 and p<0.05). In cells from HS, infection with a DL isolate also induced higher TLR expression than CL.

### Expression of Proinflammatory Cytokines

**Figure 6:**
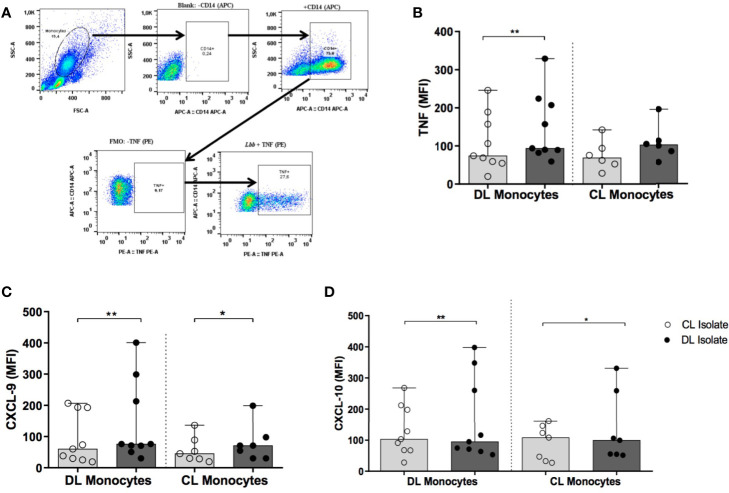
Production of TNF, CXCL9 and CXCL10 by monocytes from patients with DL and CL following infection with different *L. braziliensis* isolates: PBMC from patients with DL (n=9) and CL (n=6) were infected with different isolates of *L. braziliensis* (5:1) for 2 h Cells were treated for 6 h with Stop Golgi, followed by surface staining with an anti-CD14 antibody for monocyte characterization. After permeative treatment, cells were marked with anti-TNF, anti-CXCL9 and anti-CXCL10 antibodies for flow cytometric analysis. **(A)** Representative graph illustrating intracellular production of TNF by flow cytometry. **(B)** TNF expression **(C)** CXCL9 expression. **(D)** CXCL10 expression. Results are presented as median fluorescence intensity (MFI) values. All p values were obtained using Wilcoxon’s signed-rank testing, while comparisons between groups were made using the Mann- Whitney statistical test (*p < 0.05) (**p < 0.01).

**Figure 6** shows the median MFI values obtained from DL and CL monocytes expressing TNF, CXCL9 and CXCL10 following infection with isolates of *L. (V.) braziliensis* from DL or CL patients.

Median TNF expression (MFI) in DL monocytes infected with a DL isolate was 94 (82-224) *versus* 74 (20-246) using a CL isolate (p<0.01) (**Figure 6B**), yet similar results were not seen in CL monocytes. With regard to CXCL9 expression, higher median MFI was observed in DL cells infected with a DL isolate compared to CL: 76 (30-401) *versus* 60 (19-207) (p<0.01). In CL monocytes, median CXCL9 expression following infection with a DL isolate was 71 (30-199) *versus* 45 (20-136) for CL (p<0.05) (**Figure 6C**). Again, median levels of CXCL10 production in DL monocytes were also higher following infection with a DL isolate compared to CL: 103 (28-268) *versus* 95 (53-398) MFI, respectively (p<0.01). Similar results were obtained in monocytes from CL patients: infection with the CL isolate induced a median MFI of 99 (52-331) for CXCL10 *versus* 108 (27-161) for DL (p<0.05) (**Figure 6D**). Similarly higher levels of TNF, CXCL9 and CXCL10 were detected in supernatants of PBMCs stimulated with SLA from DL isolates in comparison with SLA from CL isolates (data not shown).

No significant differences were observed in the expression of CXCL9 or CXCL10 when comparing DL and CL monocytes infected with the same type of *L. (V.) braziliensis* isolate. Additionally, no differences were seen in IL-10 production regardless of monocyte origin (CL or DL) or the isolate used for infection (DL *vs*. CL) (data not shown).

## Discussion

DL is an emergent and severe form of *L. (V.) braziliensis* infection (Turetz et al., 2002; Machado et al., 2011; Vernal et al., 2016). While the mechanism underlying parasite dissemination has yet to be identified, it is clear that this does not occur at the time of infection. Indeed, multiple acneiform and papular lesions appear suddenly only days or weeks after a primary ulcerated lesion and dissemination is associated with fever and chills lasting 1-2 days (Machado et al., 2011). As *L. (V.) braziliensis* is polymorphic, distinct genotypic characteristics among isolates have been linked to different clinical forms of disease, e.g. cutaneous, mucosal, DL or atypical CL (Schriefer et al., 2004; Queiroz et al., 2012; Guimaraes et al., 2016). Here we attempted to evaluate whether infection with DL isolates provokes different behavior compared to CL in terms of parasite internalization and multiplication, as well as monocyte activation and the production of inflammatory molecules. The present results indicate that *L. (V.) braziliensis* isolates from DL patients exhibit a greater ability to penetrate and multiply in monocytes compared to CL, despite higher respiratory burst induction and enhanced proinflammatory cytokine production. Additionally, host factors also play a role in parasite dissemination, as monocytes from DL were found to be more permissive to infection and allowed enhanced parasite multiplication compared to CL cells infected with a DL isolate. Moreover, DL monocytes expressed more TLR2 and TLR4 than cells from CL patients infected with a DL isolate and higher levels of PGE2 upon stimulation with SLA in comparison to CL monocytes.

In the first 48 hours after *in vitro* infection with *L. (V.) braziliensis*, the percentage of infected monocytes/macrophages and the number of intracellular parasites increases, reaches a plateau and then begins to decrease (Giudice et al., 2012; Carneiro et al., 2016). Parasite internalization is observed *via* the quantification of these parameters in the first two hours of infection. After this time, the percentage of infected cells and numbers of parasites reflect the ability of leishmania to multiply inside host cells, as well the capacity of these infected cells to kill parasites. Here, we demonstrate that experimental infection using an isolate from a DL patient enables greater monocyte penetration and survival compared to an isolate from CL, regardless of the origin of source cells, i.e. DL or CL monocytes, which provides evidence of the capability of genotypic differences among *L. (V.) braziliensis* to interfere in parasite internalization and multiplication. In cells from HS, the percentage of infected cells and number of amastigotes inside cells were greater in monocytes infected with DL compared to CL isolates, yet this was not the case at all timepoints, giving support that monocyte source also influences parasite internalization and multiplication.

Parasite proliferation inside phagocytic cells leads to cell lysis and the release of *Leishmania* (Novais et al., 2009). Our results document the greater viability of the DL isolate compared to CL in the supernatants of DL, CL and HS monocyte cultures. Interestingly, and perhaps more important than genotypic differences among parasites, monocytes from DL patients were observed to allow enhanced leishmania multiplication, as higher numbers of viable promastigotes were observed following infection with a DL isolate in the supernatant of cultured DL monocytes compared to CL. This result provides evidence that DL monocytes were more permissive to leishmania survival, regardless of the source of *L. (V.) braziliensis*, i.e. isolates from DL or CL patients.

SOD enhances parasite multiplication in macrophages and is highly expressed in host tissue during CL infection by *L. (V.) braziliensis* (Khouri et al., 2009). In addition, in CL caused by *L. amazonensis*, SOD plasma levels constitute a predictor of failure to meglumine antimoniate therapy (Khouri et al., 2014). More recently, PGE-2 has been associated with leishmania proliferation and survival (Lonardoni et al., 1994; Araujo-Santos et al., 2014; Saha et al., 2014). While no differences were found herein in SOD serum levels, we did observed that, upon stimulation with SLA, monocytes from DL patients produced higher levels of PGE-2 than cells from CL patients. This finding lends support to the role of monocytes, since origin, i.e. DL *vs*. CL, as was shown to influence parasite survival.

Monocytes from CL patients present higher oxidative burst and also produce more reactive oxygen species (ROS) and nitric oxide (NO) than cells from HS; regardless, these phenomena are insufficient to control parasite multiplication and may still lead to pathology (Carneiro et al., 2016). The importance of NO in the killing of *L. infantum* and *L. amazonen*sis has been documented, as well as that of ROS in the control of *L. (V.) braziliensis* proliferation (Liew et al., 1990; Diaz et al., 2003; Novais et al., 2014). Ávila et al. showed that *L. (V.) braziliensis* promastigotes and amastigotes isolated from CL and ML patients produced similar amounts of NO in culture. However, promastigotes from ML isolates were found to be more resistant to NO and H_2_O_2_ than CL parasites (Avila et al., 2018). Our results document that infection with DL isolates induces higher respiratory burst in monocytes from DL, CL and HS compared to isolates from CL, yet the observed enhancement in oxidative burst did not inhibit parasite multiplication, which suggests that DL isolates are less susceptible to monocyte killing than CL.

While TLRs are known to participate in host defense mechanisms, the expression of TLRs has also been associated with inflammatory and autoimmune diseases (Patra et al., 2020). Our results show that monocytes from DL patients infected with DL isolates express more TLR2 and TLR4 compared to CL isolates. However, we did not similarly document this in CL monocytes. As cells from DL expressed more TLRs upon infection with DL isolates, but also exhibited a decreased capability to kill leishmania, we suggest that the exaggerated inflammatory response observed in DL may likely be more closely related to pathology than protection.

Proinflammatory cytokines, such as TNF, CXCL9 and CXCL10, are mainly produced by monocytes/macrophages. In CL patients, these cells produce comparatively higher levels of cytokines than healthy subjects (Giudice et al., 2012). Elevated systemic production of CXCL9 has been documented in sera from DL patients compared to CL (Machado et al., 2011). Additionally, upon stimulation of PBMCs from both CL and DL patients with SLA obtained from the isolate of a DL patient, higher TNF and IFN-γ expression was observed in comparison to SLA generated from a patient with CL (Leopoldo et al., 2006). The present work expanded on these observations by demonstrating that infection with a DL isolate induced comparatively higher TNF, CXCL9 and CXCL10 expression than CL in cultured monocytes obtained from both CL and DL patients. Indeed, it appears controversial that infection using DL isolates induces a pronounced proinflammatory response in monocytes, while still permitting parasite survival inside phagocytic cells. However, it is important to also consider that proinflammatory cytokine production by monocytes has not been definitively linked to parasite killing in CL caused by *L. (V.) braziliensis* (Giudice et al., 2012).

Our data indicate that parasite dissemination in DL occurs due to parasite multiplication, cell death and the release of amastigotes that infect monocytes at different sites of the skin. However, we cannot rule out the possibility that parasite dissemination may also occur through the metastasis of infected monocytes from the original lesion site to other areas of the body. In cancer, inflammation is known to influence metastasis. More specifically, the production of CXCL9 and CXCL10, among other proinflammatory cytokines, has been associated with the severity of melanoma and metastasis (Harlin et al., 2009; Lok et al., 2014). Here we expanded on a previous report that documented higher proinflammatory cytokine production induced by DL isolates compared to CL (Leopoldo et al., 2006). In light of a lack of evidence suggesting that inflammation correlates with leishmania killing in *L. (V.) braziliensis* infection, it is important to consider the possibility that inflammation may hold influence over parasite dissemination.

DL, a severe and emergent form of *L. (V.) braziliensis* infection, has been associated with high rates of failure to antimony therapy. Our results show that parasite dissemination can be influenced by host and parasite factors, and that parasite multiplication in macrophages is closely linked to parasite dissemination. As inflammation has been associated with the pathology of *L. (V.) braziliensis* infection, a combined regimen of chemotherapy with immunomodulatory agents in the treatment of tegumentary leishmaniasis (TL) should be encouraged. Moreover, macrophage reprogramming has been shown to contribute a valuable tool for tumor therapy, and reprogramming macrophages to enhance the ability to kill *Leishmania* should also be considered as an immunotherapeutic strategy to treat TL (Lessa et al., 2001; Almeida et al., 2005). In addition to offering insight into the pathogenesis of DL, the present results point to the necessity of identifying novel therapeutic agents capable of enhancing leishmania killing by macrophages to enable better control of leishmaniasis.

## Data Availability Statement

The data used to support the findings of this study have been deposited in the figshare repository (DOI: 10.6084/m9.figshare.16553394).

## Ethics Statement

The studies involving human participants were reviewed and approved by Institutional Review Board of the Federal University of Bahia, and was approved by the Brazilian Commission for Ethics in Research (CONEP) (protocol no.: 2.114.874). The patients/participants provided their written informed consent to participate in this study.

## Author Contributions 

WO, AS, OB, and EC designed the study. WO, AD, PC, and MN performed the experiments. WO, OB, LC, and AS analyzed and interpreted the data. PM participated in the diagnosis and treatment of the patients. OB and EC wrote the manuscript. All authors contributed to the article and approved the submitted version.

## Funding

This work was supported by the National Institutes of Health (AI 136032 to EC) and by the Foundation for Support Research in the State of Bahia, Brazil (FAPESB).

## Conflict of Interest

The authors declare that the research was conducted in the absence of any commercial or financial relationships that could be construed as a potential conflict of interest.

## Publisher’s Note

All claims expressed in this article are solely those of the authors and do not necessarily represent those of their affiliated organizations, or those of the publisher, the editors and the reviewers. Any product that may be evaluated in this article, or claim that may be made by its manufacturer, is not guaranteed or endorsed by the publisher.
